# Short-Term Crossover Study on the Effect of Orthogonalized Deep-Grooved Posterior Artificial Teeth on the Masticatory Efficiency of Complete Denture Wearers

**DOI:** 10.2174/1874210601812010255

**Published:** 2018-03-30

**Authors:** Yuki Hashimoto, Kyoko Sugimoto, Yuki Tanaka, Hikaru Sugimoto, Shogo Minagi

**Affiliations:** 1 Department of Occlusal and Oral Functional Rehabilitation, Okayama University, 2-5-1 Shikata-cho, Kita-ku, Okayama 700-8525, Japan; 2 Dental Education Reforming Unit, Center for the Development of Medical and Health Care Education, Okayama University, 2-5-1 Shikata-cho, Kita-ku, Okayama 700-8525, Japan

**Keywords:** Mastication, Artificial teeth, Masticatory efficiency, Elderly, Denture, Lingualized occlusion

## Abstract

**Background:**

Mastication has been regarded to play an important role in achieving quality daily life for the elderly. On the occlusal surface of posterior artificial teeth, parallel grooves of 1 mm depth and 1 mm width with an inter-groove distance of 2 mm have been found to significantly improve masticatory efficiency on a mechanical simulator.

**Materials and Methods:**

In the present study, the effect of the grooved design on masticatory efficiency in edentulous subjects using a short-term crossover trial was evaluated. Six edentulous participants, 1 male and 5 females, were assigned into two groups. One received duplicated complete dentures with grooved molars first, and the other received duplicated complete dentures with conventional molars first. The design of the teeth was crossover after evaluating masticatory efficiency. Raw carrot, raw lettuce and mixed foodstuffs were used to evaluate masticatory efficiency. Subjects were instructed to masticate 20 strokes and arbitrary strokes until they felt like swallowing.

**Results:**

The number of masticatory strokes taken was significantly smaller using the grooved design (*p*<.05). Masticatory efficiency analysis revealed that the grooved design achieved significantly higher masticatory efficiency at 20 masticatory strokes for raw carrot and mixed foodstuffs (*p*<.05).

**Conclusion:**

Results show that compared with conventional lingualized occlusion, the grooved design accomplishes higher masticatory efficiency *in vivo*. Considering the distinction of this design that it can be applied to any existing denture occlusion, the design might help improve the dietary life of the elderly.

## 1 INTRODUCTION

Functional limitations in speaking and eating as a result of tooth loss have been reported to seriously affect the quality of life (QoL) of the elderly [1]. With the increase in age, the number of masticatory strokes required to chew a standard piece of food also progressively increases. Additionally, less comminution is achieved in a longer chewing sequence. Thus, denture prostheses with higher masticatory efficiency have been in great demand to improve the QoL of the elderly [2]. Although improved mastication in edentulous patients has been shown using implant prostheses [3], conventional complete denture prosthesis remains the most common treatment for edentulous patients [4]. Questionnaire studies on food consumption of elderly subjects revealed that tooth loss leads to dietary modifications because people choose foods that are easier to chew [5]. Many kinds of occlusal surfaces and the functional differences among them have been reported to date [6-8]. Considering the rapid increase of aged populations, it would be beneficial to establish a method that could improve the masticatory efficiency of complete dentures not only in and of themselves, but even when being used while actually eating [9]. Hashimoto *et al*. recently reported a new groove design for the occlusal surface of dentures that could be applied to any conventionally designed occlusal scheme [10]. They reported that this groove design showed significantly higher masticatory efficiency compared with conventional lingualized occlusion when evaluated on experimental upper and lower complete dentures mounted on a masticatory simulator. Although their design is inherent of great merit in that it can be applied to any currently used denture prosthesis, the effect remains to be proved clinically.

The present study aims to evaluate the effect of the grooved design on masticatory function in edentulous subjects using a crossover design trial.

## 2 MATERIALS AND METHODS

This paper report results from a single center, short-term crossover trial [11]. The trial protocol was approved by the Ethics Committee of Okayama University (#2200).

### 2.1 Participants

The present study was conducted from September 2015 to February 2016 in the clinical division of removable prosthodontics at Okayama University Hospital. Six participants, 1 male (aged 82 years) and 5 females (aged 80.4 ± 3.6 years), who were edentulous in both arches and currently using complete dentures (CDs) that were fabricated in Okayama University Hospital for more than 1 month participated in this study. All participants gave informed consent and signed a letter of consent.

### 2.2 Study Design

This study design is a short-term crossover randomized controlled clinical trial [11], implying that all study participants were subjected to two consecutive test conditions with varying occlusal surface designs of the posterior teeth (grooved versus non-grooved). The sequence of the test conditions was randomized for each participant by YH. Three participants were assigned to group A, receiving upper and lower copy dentures with grooved molars first. Another three participants were assigned to group B, receiving upper and lower copy dentures with conventional design molars first. Prior to the study, all participants confirmed that they had been using their dentures comfortably for >1 month. Conventional lingualized occlusion was adopted as the occlusion scheme in all participants. For each participant, precision-duplicated CDs of their currently used dentures were fabricated. On the occlusal surface of the molars of the duplicated dentures of group A, parallel grooves were prepared using a diamond disc (HORICO disc, Mokuda Dental Co., Ltd., Kobe, Japan) according to Hashimoto *et al*. groove design [10]. In brief, the width of each groove was 1 mm and the inter-groove distance was 2 mm. The depth of the groove was 1 mm at the buccolingual center of each artificial molar tooth, and 0 mm at the buccal and lingual edges of the occlusal surface. Fig. (**1**) shows one example of a clinical case of dentures with grooved teeth. The parallel grooves were prepared at a 45° angle to the dental arch, as shown in Fig. (**1**). The direction of grooves on the maxillary molars was designed to be orthogonally oriented towards those on the mandibular molars when occluded. The duplicated dentures were used for 1 week before evaluating masticatory efficiency. After this 1 week period, the first session to evaluate masticatory efficiency was done. Subsequently, the occlusal surface design was modified. Modification of the grooved artificial teeth group A involved using composite resin (CLEARFIL AP-X Flow, Kuraray Noritake Dental Inc., Tokyo, Japan) to fill the groove to restore the original occlusal surface. Conversely, modification of the artificial teeth of group B involved preparing a 1-mm width groove as described above. Careful attention was paid to ensure the occlusal contact points in the intercuspal position were not altered, and likewise to ensure the gliding paths of the cusps on molars were not altered. Participants confirmed that they did not feel any perceptible change to occlusion after the modification. Then, a second session to evaluate masticatory efficiency was conducted.

### 2.3 Evaluating Masticatory Efficiency

Considering the purpose of evaluating masticatory efficiency in this study, objectively evaluating actual daily dietary life is ideal. To address this aim as faithfully as possible, image analysis of food particles using a mouthful of mixed foodstuffs, which simulated an actual meal, was done according to Sugimoto *et al*. method [12].

Each participant was instructed to take one mouthful of mixed foodstuffs comprising cooked rice (2.9-3.1 g), one piece of round-sliced sausage (0.5-cm-thick, 1.5 cm in diameter, 1.4-1.7 g), one piece of cuboid Tamagoyaki (Japanese hard omelet, 0.7-1.0 g, 1.0 × 1.0 × 0.5 cm), julienne strips of raw cabbage (0.3-0.4 g) and one piece of round-sliced raw cucumber (0.5-cm-thick, ca. 1.5 cm in diameter, 2.4-3.0 g). Thus, a total of 8.6 ± 0.5 g was masticated as one mouthful. In addition, two other experimental foods were used: three pieces of raw lettuce leaf of (3.0 × 1.0 cm), and three pieces of cuboid cut raw carrot of (1.0 × 1.0 × 1.0 cm). Before the evaluation procedure, as a practice session, participants were allowed to freely masticate and swallow each of the three experimental foods. At the evaluation stage, for each of the three experimental foods, participants were instructed to masticate 20 masticatory strokes first, then the food boluses were retrieved in a plastic cup. Participants were then instructed to masticate another set of each food freely until they were likely to swallow. The boluses were retrieved just before swallowing in plastic cups. The number of masticatory strokes at which participants were likely to swallow each food was recorded. Food particle analysis for each bolus was achieved using a masticatory efficiency evaluation system (SME-002; Shofu, Inc., Kyoto, Japan). Evaluation of masticatory efficiency was triplicated for each mastication condition. The food particle size index (SI) and homogeneity index (HI) were automatically calculated for a sample bolus in the masticatory efficiency evaluation system. Smaller HI values indicate higher homogeneity to observed particle size, and smaller SI values indicate that food was masticated into smaller particles [13]. HI value <0.1 and SI value <1.62 were reported to be the thresholds for normal mastication ability in healthy young adults, regardless of the food material masticated [12].

### 2.4 Statistics

Wilcoxon signed-rank test was used to evaluate the difference between two occlusal surface designs. Data analyses were performed using *SPSS* software (IBM *SPSS* Statistics version 20.0; IBM Corp., Armonk, NY, USA). A significance level of *p* < 0.05 was used in this study.

## 3 RESULTS

The number of masticatory strokes taken when participants felt like swallowing is summarized in Fig. (**2**). The number of masticatory strokes taken was significantly smaller in the G+ condition than that for G- in all three experimental foods, except for one participant (*p*<.05). Participant #2 showed an exceptionally increased number of masticatory strokes taken for lettuce in the G+ condition. Upon finishing all trials, she revealed that she let slip the lettuce sample in one instance. The mean number of masticatory strokes taken for carrot under G- and G+ conditions were 62.1± 9.5 and 49.5± 10.9, respectively. The mean reduction in the number strokes taken when using the groove design for carrot was 20.3%. The mean number of masticatory strokes taken for lettuce under G- and G+ conditions were 37.1± 7.5 and 29.3± 9.2, respectively. The mean reduction of the number of strokes taken when using the groove design for lettuce was 21.0%. The mean number of masticatory strokes taken for lettuce under G- and G+ conditions were 37.1± 7.5 and 29.3± 9.2, respectively. The mean reduction in the number strokes taken when using the groove design for lettuce was 21.0%. The total mean number of masticatory strokes taken for all three test foods under G- and G+ conditions were 53.1± 15.0 and 41.2± 12.5, respectively. The mean reduction in the number of strokes taken when using the groove design for the total test foods was 22.4%. Although 3 participants gave up on comminuting raw carrot under the G- condition because of pain caused by the mandibular denture to supporting mucosa, 2 of these participants could comminute raw carrot under the G+ condition.

**Fig. (3):**
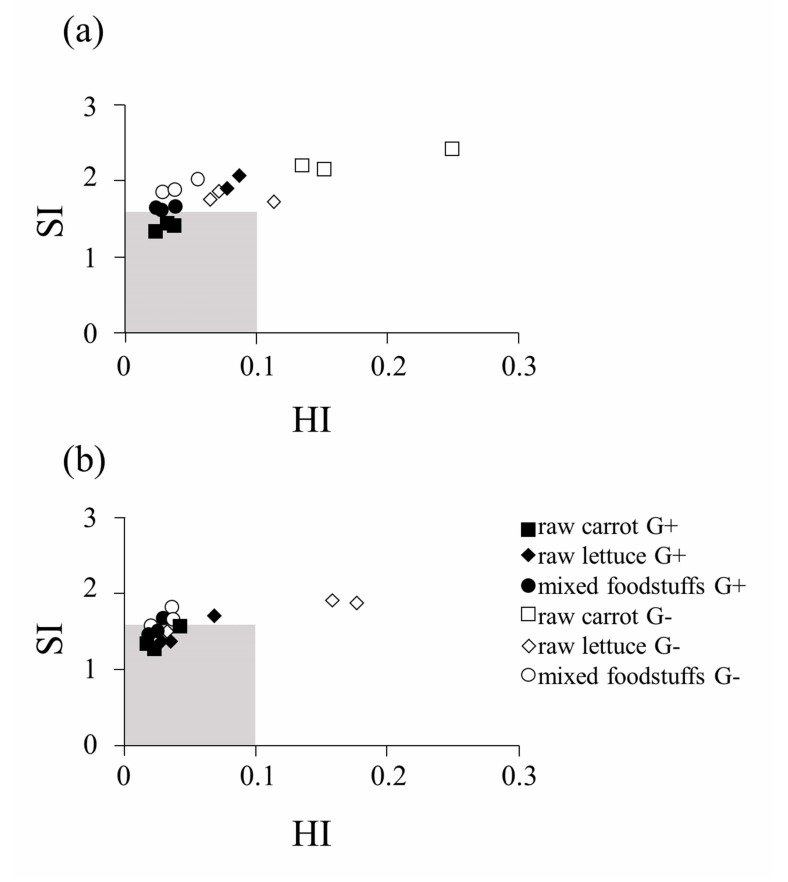
Typical data examples of SI and HI values. (a) masticated for 20 masticatory strokes (b) masticated until just before natural swallowing. Shaded area shows the range of normal mastication reported by Sugimoto *et al*. [13]. Note that closed dots (G+) locates closer to the shaded range than open dots (G-) at 20 masticatory strokes.

Typical data evaluating masticatory efficiency (participant #5) is shown in Fig. (**3**). The shaded areas on Fig. (**3**) show the normal masticatory range (HI<0.1 and SI<1.62) reported by Sugimoto *et al*. [12]. In this participant, comminution without grooves did not reach the normal masticatory range at 20 strokes of mastication as shown in Fig. (**3a**). However, most samples masticated using grooved artificial teeth were close to the normal masticatory range and some were within the range at 20 strokes. Just before natural swallowing, most comminutions of the samples were closer to the range regardless of groove design (Fig. **3b**). Therefore, G+ design resulted in finer comminution with a smaller number of masticatory strokes than that for the G- design in this participant.

The HI and SI at 20 masticatory strokes are shown in Fig. (**4**). For raw carrot and mixed foodstuffs, significantly smaller values of HI and SI were observed in the G+ condition compared with that for G- (*p*<.05). Although no statistical significance was shown, a similar tendency was observed for raw lettuce.

The results of particle analysis of the boluses retrieved just before swallowing are shown in Fig. (**5**). No significant differences were observed for G- and G+ conditions. However, in the G+ condition, all mean and median values of HI and SI were below the threshold for normal mastication. Conversely, in the G- condition, the mean and median values of HI and SI for raw carrot and raw lettuce were above the threshold of normal mastication.

## 4 DISCUSSION

The relationship between dental status and diet has been an issue of recent interest. Nowjack-Raymer and Shelham reported that people with fewer than 28 teeth had significantly lower intakes of carrots, tossed salads, and dietary fiber than did fully dentate people, as well as lower serum levels for beta-carotene, folate, and vitamin C, showing that dental status significantly affects diet and nutrition [14]. Hung *et al*. reported in their epidemiological questionnaire study on 83,104 US women that women with fewer teeth have unhealthier diets such as decreased intake of fruits and vegetables, which could increase their risk of developing cardiovascular diseases [15]. The often observed difficulty of edentulous people in masticating fibrous foods, fruits and vegetables could contribute to these conditions. Therefore, fabrication of CD with higher masticatory efficiency would be of great importance for not only improving these conditions but also improving QoL of the elderly.

Changing the occlusal form or material of artificial posterior teeth is one important and simple way of improving masticatory efficiency. Metal-bladed teeth are a widely recognized support for posterior teeth [16] . Metal-bladed teeth have excellent cutting properties and would effectively cut through the meat. However, the bladed teeth, in principle, are not suitable for levigating a food bolus because of its limited occluding surface area. Elderly or super-elderly people are often served soft or chopped foods because their deglutition ability is usually low [17]. Therefore, high efficiency of levigating foods would be expected for the posterior teeth of CDs in the elderly. High masticatory efficiency for raw fibrous vegetables would also be expected. To satisfy the aforementioned requirements for the dietary life of the elderly, the artificial posterior teeth would need to fulfill the following:

1. Considering the high prevalence of a resorbed ridge with low ability to bear occlusal force, high cutting efficiency is required to reduce possible pain on denture-supporting tissues.
2. For effective levigation of soft foods, a wide occlusal table area with wide opposing teeth is necessary.
3. For easy mastication of raw vegetables such as lettuce, shredding function should be effective.
4. Applicability of the posterior teeth to any existing occlusal schema, including full-balanced occlusion or lingualized occlusion, is necessary.

Hashimoto *et al.* established a novel design of posterior teeth that would fulfill these requirements using the masticatory simulator system [10]. They revealed the occlusal surface design using parallel grooves of 1-mm depth, 1-mm groove width and 2-mm inter-groove distance showed highest masticatory efficiency. The advantages of this design are as follows:

1. Groove design increases the force per unit area exerted to foodstuffs without reducing the surface area of the occlusal table, thereby reducing the load on denture-supporting tissues.
2. The orthogonal grooves of the upper and lower occlusal surfaces would ensure shredding force during grinding movement.
3. As the upper and lower grooves cross orthogonally when occluded, they do not get stuck during grinding movement.
4. As the grooves can be easily ground into any artificial posterior teeth, the design could be applied to any existing dentures.

The mean number of masticatory strokes observed in this study without grooves was 53.1±15.0. Toda *et al.* reported that the number of masticatory strokes taken until swallowing ranged from 23.3±10.6 to 60.1±38.1 depending on food material [18]. The mean number of masticatory strokes observed for non-grooved teeth in this study well agree with their data, suggesting the relevance of the controlled setting of this study. Prinz and Lucas reported that the number of chews made before swallowing was significantly affected by particle size and also by the concentration of large particles in food samples, suggesting that food is swallowed only when particles are both small enough and sufficiently lubricated [19]. The mean number of masticatory strokes taken before swallowing was shown to be reduced by >20% when the groove design was applied, regardless of the food material used. Morizawa *et al*. studied the effect of occlusal scheme on the number of masticatory strokes taken before swallowing, focusing on the difference between the full balanced occlusion and lingualized occlusion, and reported that the difference was 3.3±1.9% [20]. The mean reduction in the number of masticatory strokes taken in the G+ condition was 22.4% compared with that for the G- condition, the conventional lingualized occlusion. Therefore, from these findings, it would be reasonable to interpret that the groove design would be worth applying to any CD used by the elderly. In this study, the objective index of the size of a food particle, SI, showed the significant effect of the groove design on reducing particle size compared with non-grooved conventional lingualized occlusion. However, aside from the simple geometrical size evaluation (SI), the reduction in the number of masticatory strokes added another physiological aspect to the effect of the groove. The reduction in the number of masticatory strokes inherently means that the comminution level achieved by the groove design surpassed the threshold of CD wearers’ oral sensation to allow swallowing. This would be of great clinical importance. This drastic reduction in a number of masticatory strokes would effectively reduce the forced burden on the denture-supporting tissues during a meal.

Comparing the data at 20 masticatory strokes in the G+ condition and the data just before swallowing in the same condition, we derived that the mean and median HI and SI for all food materials proceeded into the normal mastication threshold according to a number of mastication strokes taken after the initial 20 strokes. Conversely, in the G- condition, the mean and median HI and SI for raw carrot and raw lettuce remained at similar values even after many strokes after the initial 20 strokes to just before swallowing, suggesting that the comminution did not effectively proceed as that for the G+ condition. From these findings, artificial teeth with grooves showed higher masticatory efficiency than those without grooves not only for the initial break down of the food material but also for the later comminution process to divide the food into finer particles.

The present study has several limitations. Although we adopted a short-term crossover design that appeared to eliminate selection bias well, the small number of participants would be one study limitation. Future studies with larger sample sizes are recommended. In the present study, the wash-out period was not set in the study design. This was done to eliminate additional possible adaptation of the participant to the duplicate denture during the wash-out period. The only difference between these two experimental denture conditions was the ground groove on the occlusal surface. To illustrate, the shape of the denture base, the position of the artificial teeth, and the occlusal vertical height were completely the same between these two conditions. Therefore, we did not adopt a wash-out period. However, from the perspective of study design, omitting the wash-out period might be regarded as one study limitation.

From the results of the present study, CDs with artificial posterior teeth that have parallel grooves of 1-mm depth, 1-mm width and inter-groove distance of 2 mm showed higher masticatory efficiency than CDs with conventional lingualized occlusion.

## CONCLUSION

The present study showed that the grooves on the occlusal surface of artificial posterior teeth of 1-mm depth, 1 mm width with an inter-groove distance of 2 mm improve masticatory performance. Considering the distinction of this design that it can be applied to any existing denture occlusion, the design might help improve the dietary life of the elderly.

## Figures and Tables

**Fig. (1):**
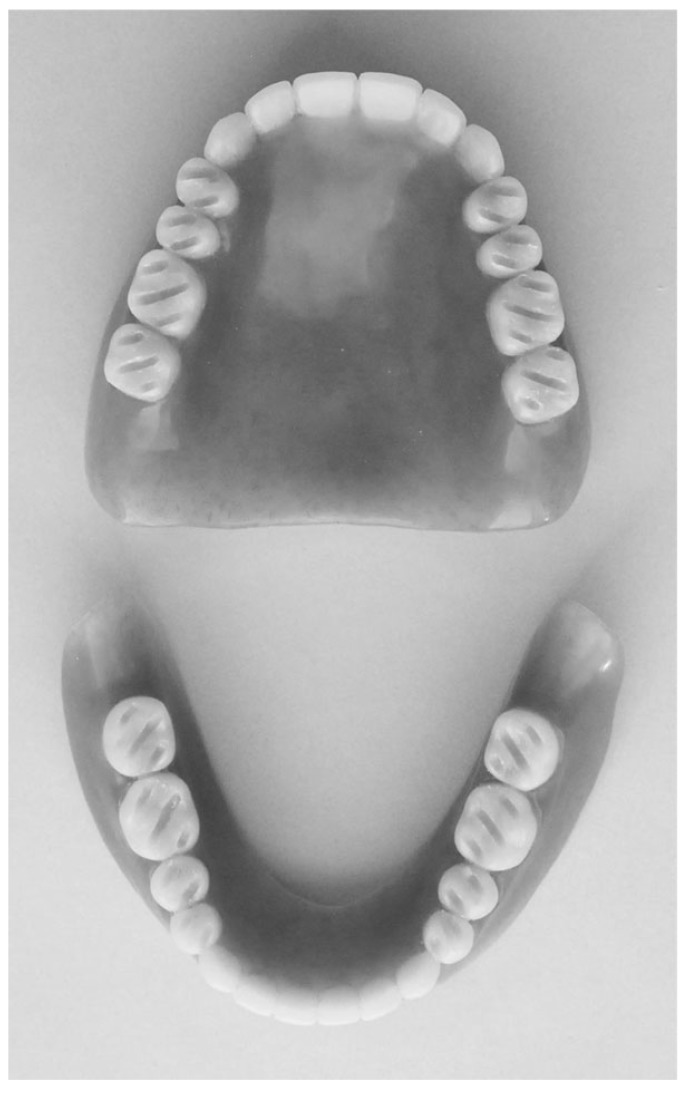
Experimental denture with grooves on artificial posterior teeth.

**Fig. (2):**
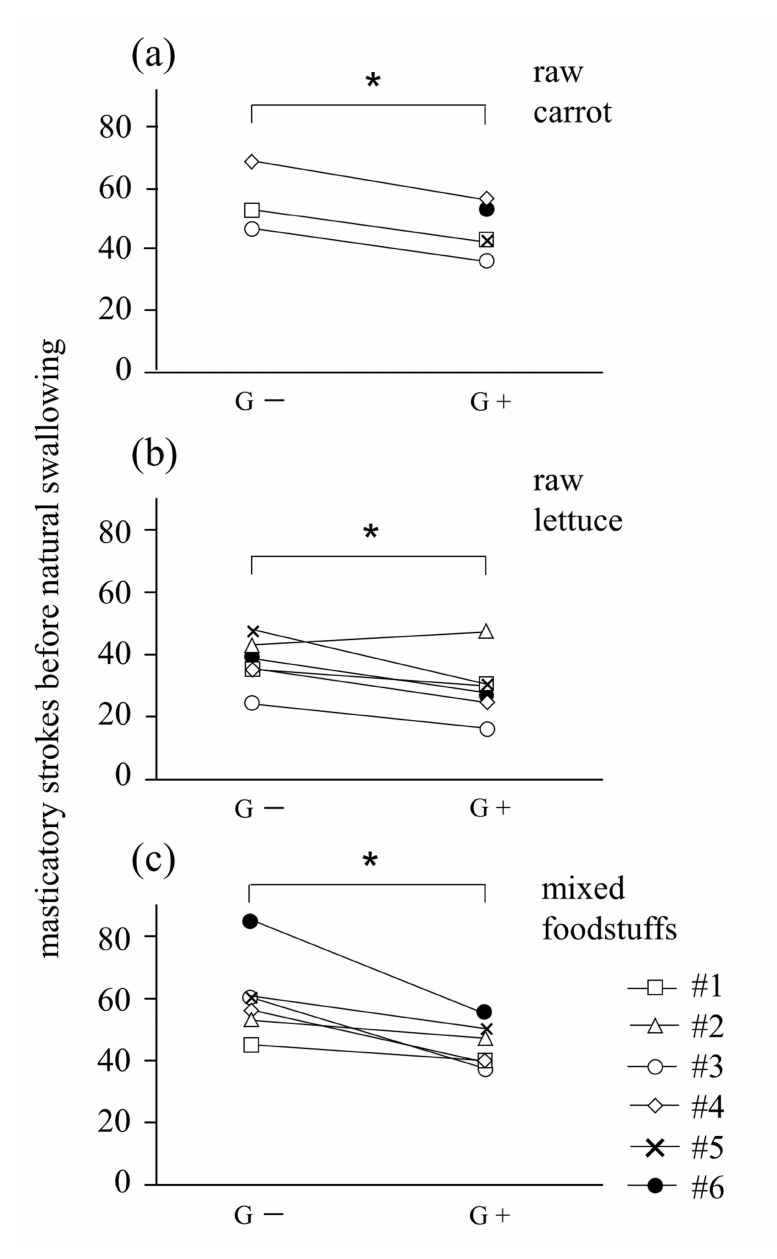
Number of chewing strokes necessary before natural swallowing. (a) raw carrot (b) raw lettuce (c) mixed foodstuffs for the number of masticatory strokes of each subjects. Note that the number of masticatory strokes for G+ was significantly smaller than G- in each of three experimental food materials (p<.05).

**Fig. (4):**
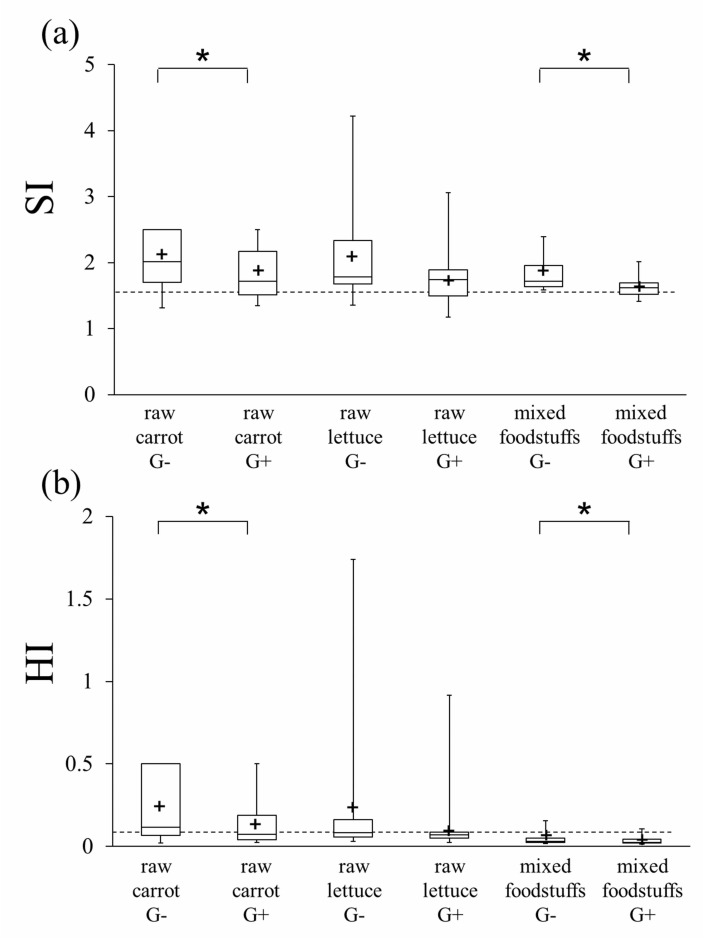
Box-and-whisker plot for HI and SI values for 20 masticatory strokes of all the subjects. The dotted lines show the thresholds for the range of normal mastication for HI (0.10) and SI (1.62) reported by Sugimoto *et al*. [13]. SI and HI values for raw carrot and mixed foodstuffs with G+ were significantly lower than those with G- (*p*<.05). +, mean value.

**Fig. (5):**
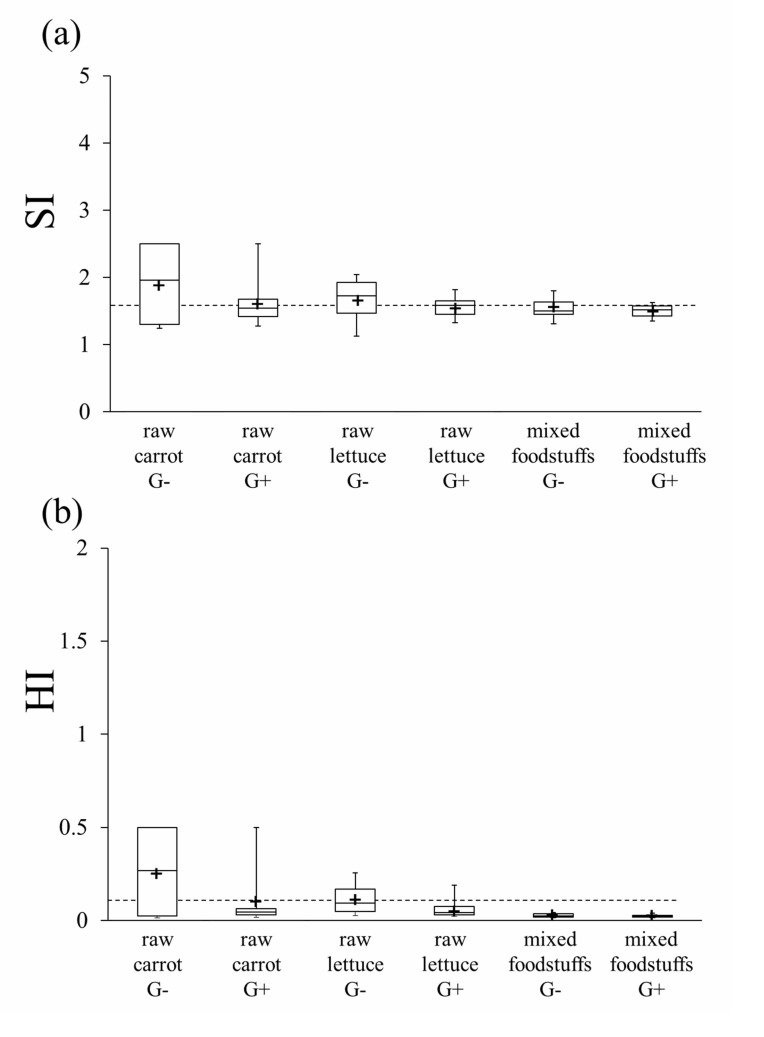
Box-and-whisker plot for HI and SI values of all the subjects just before swallowing. The dotted lines show the thresholds for the range of normal mastication for HI (0.10) and SI (1.62) reported by Sugimoto *et al*. [13]. Note that the median value for G+ for all the experimental food condition are lower than the threshold. +, mean value.
